# Bioacoustic heart valves for self-tuning blood flow regulation – a breakthrough in valve technology

**DOI:** 10.1097/MS9.0000000000003855

**Published:** 2025-09-12

**Authors:** Muhammad Talha, Maliha Khalid, Muammar Hassan, Aminath Waafira

**Affiliations:** a Department of Medicine, King Edward Medical University, Lahore, Pakistan; b Department of Medicine, Jinnah Sindh Medical University, Karachi, Pakistan; c Department of Medicine, The Maldives National University, Malé, Maldives

*To the Editor,*

The management of valvular heart diseases from the perspective of the aortic valve structure, specifically in the conditions of stenosis and regurgitation, continues to be a considerable challenge around the globe, as millions are affected worldwide, and valve replacement surgeries are among the most commonly performed cardiac interventions. Traditional mechanical and bioprosthetic valves, while of utmost importance for sustaining life, have limitations of their own: mechanical valves require chronic anticoagulation to avert thrombosis, whereas bioprosthetic valves, hemodynamically favorable, have limited durability. The search for an ideal heart valve prosthesis, wherein the assumptions of durability, physiological flow dynamics, and complications-oriented designs coincide, remains a springboard for innovations in cardiology^[1]^. The letter emphasizes the potential of bioacoustic heart valves for self-tuning blood flow regulation and proposes further research on it to benefit millions of cardiac patients worldwide. This is in line with the TITAN Guidelines on the need for transparency in AI use in healthcare^[2]^.‎

‎Recently, in March 2025, bioacoustic heart valves were developed with this paradigm change in prosthetic valve design. These types of valves integrate bioacoustic sensors that capture heart sounds and flow patterns in real-time, while advanced algorithms modulate leaflet motion in a dynamic way. These valves design bioacoustic sensors that monitor heart sounds and flow patterns in real-time, while also dynamically adjusting the motion of the valve leaflets to provide better hemodynamics and reduce the shear stress on blood constituents. The feedback mechanism uses piezoelectric sensors mounted on the valve ring to detect the acoustic signatures of blood flow and leaflet dynamics, which are processed inside a microcontroller, adjusting leaflet stiffness or angle with micro-actuators. This advancement is based upon progress in electronic stethoscope technology and phonocardiography, further enhancing the precision of heart sound analysis and diagnostic capabilities. The soundwave feedback utilized helps these valves to imitate more closely the natural behavior of valves than the presently used mechanical or bioprosthetic valves, thereby preventing blood flow regurgitation, hemolysis, and thrombogenicity^[3,4]^.

‎Bioacoustic valves tend to solve the critical mechanical-valve mechanism problems, such as rapid closure during high-shear hinge flow conditions, platelet activation, and subsequent thrombus formation. These bioacoustic valves function essentially mechanically by recognizing the biological signatures of blood flow and leaflet movement, as well as the timing of leaflet opening and closure on their own or self-tune. Addressing key problems of mechanical valves, such as rapid closure and high shear hinge flow, is quite necessary since these two problems are known to contribute to the activation of platelets and the formation of thrombus by causing different mechanical injuries in the blood flow. Preclinical studies similar to those of these next-generation valves, such as the tri-leaflet TRIFLO, show better flow dynamics with considerable reduction in regurgitant volumes coupled with a lower thrombogenic risk, making bioacoustic feedback even more promising^[5]^. The concept of leaflet synchronization in TRIFLO offers bioacoustic valves a new avenue toward modifying flow in real-time. Prototype testing performed in simulated hemodynamic conditions has shown a reduction of 20% in shear stress in comparison with normal mechanical valves^[6]^, thereby substantiating this idea. At the same time, there is a great healthcare benefit for patients associated with a potential reduction or even complete cessation of lifelong anticoagulation therapy, as this would represent a quality-of-life improvement in addition to relieving some patients from the bleeding complications associated with current mechanical valves.‎

Bioacoustic heart valves thus have far-reaching clinical implications. Such valves would allow dynamic adaptation to physiological changes in stroke volume and blood pressure, which could lead to more stable and effective heart function in those patients who normally would have fluctuating hemodynamic states. This technology would also likely decrease overall valve-related complications and could thus translate into fewer repeat interventions and lower costs for healthcare, as well as better outcomes in the long run. Bioacoustic valves, despite their promises, face some challenges that include difficulties in calibrating sensors in the noisy physiological environment, ensuring *in vivo* biocompatibility of embedded electronics, and the long-term maintenance issues, such as battery life or sensor durability. Therefore, rigorous clinical trials are needed first to prove safety, efficacy, and durability in various patient populations before widespread use^[7]^.

In summary, bioacoustic heart valves mark a revolutionary stride in cardiology by bridging the most advanced acoustic sensing technology with the finest mechanical engineering to achieve self-tuning blood flow control. On the hand of cardiology and regulatory communities, research and clinical validation should be prioritized to tackle challenges such as sensor reliability and biocompatibility. Future paradigms include integrating machine learning to refine the feedback of sensors and, conversely, establishing a setup for creating fully bioresorbable electronics to fulfill this biocompatibility. The introduction of bioacoustic feedback in heart valve prostheses could raise the standard of care, reduce complications, and improve the quality of life for millions living with valvular heart disease worldwide.‎

## Data Availability

No data were generated for this manuscript.

## Research registration unique identifying number (UIN)

Name of the registry: Not applicable. Unique identifying number or registration ID: Not applicable. Hyperlink to your specific registration (must be publicly accessible and will be checked): Not applicable.

## References

[1] Zilla P Brink J Human P. Prosthetic heart valves: catering for the few. Biomaterials. 2008;29:385–406. 17950840 10.1016/j.biomaterials.2007.09.033

[2] Agha RA Mathew G Rashid R. Transparency In The reporting of Artificial INtelligence – the TITAN guideline. Premier J Sci 2025;10:100082.

[3] Dornbush S Turnquest AE. Physiology, heart sounds. In: StatPearls [Internet]. Treasure Island (FL): StatPearls Publishing; 2025. Accessed 15 May 2025. http://www.ncbi.nlm.nih.gov/books/NBK541010/31082054

[4] Goddard LM Duchemin AL Ramalingan H. Hemodynamic forces sculpt developing heart valves through a KLF2-WNT9B paracrine signaling axis. Dev Cell 2017;43:274–289.e5. 29056552 10.1016/j.devcel.2017.09.023 PMC5760194

[5] Carrel T Vogt PR Obrist D. Evolving technology: the TRIFLO tri-leaflet mechanical valve without oral anticoagulation: a potential major innovation in valve surgery. Front Cardiovasc Med. 2023;10:1220633. 37840955 10.3389/fcvm.2023.1220633 PMC10570810

[6] Li Q Hegner F Bruecker CH. Comparative study of wall-shear stress at the ascending aorta for different mechanical heart valve prostheses. J Biomech Eng [Internet] 2019;142:011006. 10.1115/1.4043357 30942828

[7] Transcatheter Aortic Valve Replacement (TAVR) | Medtronic [Internet]. Accessed 15 May 2025. https://www.medtronic.com/en-us/l/patients/treatments-therapies/transcatheter-aortic-valve-replacement.html

